# The Phosphoinositide 3-Kinase Isoform PI3Kβ Regulates Osteoclast-Mediated Bone Resorption in Humans and Mice

**DOI:** 10.1002/art.38660

**Published:** 2014-07-28

**Authors:** Dávid Győri, Dániel Csete, Szilvia Benkő, Suhasini Kulkarni, Péter Mandl, Csaba Dobó-Nagy, Bart Vanhaesebroeck, Len Stephens, Phillip T Hawkins, Attila Mócsai

**Affiliations:** 1 Semmelweis University School of Medicine, and MTA-SE “Lendület” Inflammation Physiology Research Group of the Hungarian Academy of Sciences and Semmelweis UniversityBudapest, Hungary; 2 University of DebrecenDebrecen, Hungary; 3 Babraham InstituteCambridge, UK; 4 Medical University of ViennaVienna, Austria; 5 Semmelweis University School of DentistryBudapest, Hungary; 6 Queen Mary University of LondonLondon, UK

## Abstract

**Objective:**

While phosphoinositide 3-kinases (PI3Ks) are involved in various intracellular signal transduction processes, the specific functions of the different PI3K isoforms are poorly understood. We have previously shown that the PI3Kβ isoform is required for arthritis development in the K/BxN serum–transfer model. Since osteoclasts play a critical role in pathologic bone loss during inflammatory arthritis and other diseases, we undertook this study to test the role of PI3Kβ in osteoclast development and function using a combined genetic and pharmacologic approach.

**Methods:**

The role of PI3Kβ in primary human and murine osteoclast cultures was tested with the PI3Kβ-selective inhibitor TGX221 and by using PI3Kβ^−/−^ mice. The trabecular bone architecture of PI3Kβ^−/−^ mice was evaluated using micro–computed tomography and histomorphometric analyses.

**Results:**

The expression of PI3Kβ was strongly and specifically up-regulated during in vitro osteoclast differentiation. In vitro development of large multinucleated osteoclasts from human or murine progenitors and their resorption capacity were strongly reduced by the PI3Kβ inhibitor TGX221 or by the genetic deficiency of PI3Kβ. This was likely due to defective cytoskeletal reorganization and vesicular trafficking, since PI3Kβ^−/−^ mouse multinucleated cells failed to form actin rings and retained intracellular acidic vesicles and cathepsin K. In contrast, osteoclast-specific gene expression and the survival and apoptosis of osteoclasts were not affected. PI3Kβ^−/−^ mice had significantly increased trabecular bone volume and showed abnormal osteoclast morphology with defective resorption pit formation.

**Conclusion:**

PI3Kβ plays an important role in osteoclast development and function and is required for in vivo bone homeostasis.

Phosphoinositide 3-kinases (PI3Ks) comprise a diverse family of lipid kinases involved in nearly all cellular functions as well as various disease processes ranging from cancer to metabolic and inflammatory diseases (1–6). The best known mammalian PI3Ks are the class I PI3K family members PI3Kα, PI3Kβ, PI3Kγ, and PI3Kδ (2,7). PI3Kα, PI3Kβ, and PI3Kδ mainly relay signals downstream from receptor or nonreceptor tyrosine kinases, whereas PI3Kγ is primarily involved in signal transduction by the βγ subunits of certain G protein–coupled receptors (2,7).

The functional role of PI3Ks had initially been addressed by using general PI3K inhibitors such as wortmannin or LY294002. However, more recent studies using genetic deletion approaches and the recent development of isoform-specific PI3K inhibitors have revealed highly specific functions of the different isoforms in certain biologic processes, promising novel therapeutic strategies for various disease states (2,4).

We previously demonstrated that PI3Kβ is required for arthritis development in the K/BxN serum–transfer model (8). Since that model mimics the myeloid cell–mediated effector phase of arthritis, PI3Kβ was most likely required in one (or more) myeloid lineage cell types.

Osteoclasts are highly specialized bone-resorbing cells of myeloid hematopoietic cell origin (9–11) and are responsible for basal bone resorption as well as pathologic bone loss during inflammatory arthritis, bone metastasis, and postmenopausal osteoporosis. Their role in inflammatory arthritis is indicated by reduced arthritis-induced local bone resorption upon genetic or pharmacologic blockade of osteoclasts, both in experimental mice (12–14) and in patients with rheumatoid arthritis (15).

Osteoclast development and function are directed by a number of extracellular cues including macrophage colony-stimulating factor (M-CSF), RANKL, β3 integrin–mediated adhesive interactions, and immunoreceptor-like activation signals (9–11,16,17). Wortmannin and LY294002 inhibit both the development and the resorptive activity of osteoclasts (18–21). However, the role of the different PI3K isoforms in osteoclast development and function is poorly understood.

These issues prompted us to analyze the role of PI3Kβ in primary in vitro osteoclast cultures and in in vivo bone homeostasis, using a combined genetic and pharmacologic approach. Our results indicate that PI3Kβ plays a major role in osteoclast development, osteoclast-mediated bone resorption, and in vivo bone homeostasis, likely due to its participation in the organization of the osteoclast cytoskeleton and the release of intracellular vesicles.

## MATERIALS AND METHODS

### Animals

*Pik3cb*^tm1.1Bvan/tm1.1Bvan^ (PI3Kβ^−/−^) mice carrying a homozygous deletion of exons 21–22 of *Pik3b*, the gene encoding the p110β catalytic subunit of PI3Kβ, were described previously (22) and were maintained in heterozygous form on a mixed C57BL/6:129Sv genetic background (backcrossed to C57BL/6 for ∼4 generations). Age- and sex-matched wild-type controls (mostly littermates) were obtained from the same colony. Transgenic mice ubiquitously expressing enhanced green fluorescent protein (EGFP)–tagged Lifeact (23) were provided by Dr. Michael Sixt (Institute of Science and Technology, Klosterneuburg, Austria) and were crossed with PI3Kβ^+/+^ mice to obtain Lifeact–EGFP–expressing PI3Kβ^−/−^ mice. For pharmacologic studies, C57BL/6 mice were purchased from Charles River.

Due to the limited availability of PI3Kβ^−/−^ mice, bone marrow cells for most in vitro osteoclast and macrophage cultures were obtained from bone marrow chimeras generated by transplanting PI3Kβ^−/−^ (and parallel wild-type control) mouse bone marrow cells to lethally irradiated recipients, as previously described (24,25). No differences have been observed between osteoclast cultures derived from intact mice and those derived from corresponding bone marrow chimeras of either genotype (data not shown).

Mice were kept in individually sterile ventilated cages (Tecniplast) in a conventional facility. All animal experiments were approved by the Semmelweis University Animal Experimentation Review Board.

### In vitro culture and resorption assays

In vitro osteoclast cultures were performed essentially as previously described (26). Wild-type or PI3Kβ^−/−^ mouse bone marrow cells were first cultured in the presence of 10 ng/ml mouse M-CSF (PeproTech) for 2 days. Nonadherent cells (referred to as myeloid progenitors) were then plated at 2 × 10^5^ cells/cm^2^ and cultured in the presence of 20 or 50 ng/ml recombinant mouse M-CSF and 20 or 50 ng/ml mouse RANKL (both from PeproTech) with media/cytokine changes every 2 days. Osteoclast morphology was tested 3 days later, using a commercial tartrate-resistant acid phosphatase (TRAP) staining kit (Sigma) and imaged using a Leica DMI6000B inverted microscope, and the number of osteoclasts (defined as TRAP-positive cells with ≥3 nuclei) was counted manually. The diameter of the cells was determined using ImageJ software (National Institutes of Health). For in vitro resorption assays, osteoclasts were cultured under similar conditions for 11 days on an artificial hydroxyapatite layer (BD BioCoat Osteologic slides) or on bovine cortical bone slices (Immunodiagnostic Systems), then processed according to the manufacturer's instructions, followed by imaging and determination of the resorbed area using ImageJ software.

For osteoblast–osteoclast coculture experiments, calvariae of euthanized neonatal wild-type mice were digested by 0.1% collagenase and 0.25% trypsin–EDTA (both from Sigma) in 5 consecutive rounds. The cells isolated during the last 3 rounds were plated at 10^5^ cells/well in 96-well plates and cultured for 2 days in the presence of 10 n*M* 1,25-dihydroxyvitamin D_3_ and 10 n*M* dexamethasone (both from Sigma). Wild-type or PI3Kβ^−/−^ mouse bone marrow cells were then seeded onto the osteoblasts at 5 × 10^4^ cells/well and cultured for 10 days with media changes every 2 days. TRAP expression was then determined as described above. Wild-type and PI3Kβ^−/−^mouse macrophages were generated by culturing myeloid progenitors in the presence of 50 ng/ml M-CSF without the addition of RANKL.

Human osteoclasts were differentiated from peripheral blood mononuclear cells (PBMCs) from healthy volunteers. PBMCs were obtained by dextran sedimentation and centrifugation through Ficoll-Paque (GE Healthcare) as previously described (27). Mononuclear cells were washed and plated at 2 × 10^5^ cells/cm^2^ to 24-well tissue culture plates or BD BioCoat Osteologic slides and cultured in the presence of 20 or 50 ng/ml recombinant human M-CSF and 20 or 50 ng/ml human RANKL (both from PeproTech) for 14 days with media/cytokine changes every 2 days. TRAP staining and resorption assays were performed as described above. Experiments on human cells were approved by the Semmelweis University Regional and Institutional Committee of Science and Research Ethics.

For inhibitor studies, wortmannin (Sigma) and TGX221 (Cayman Chemical) were added either concomitantly with the intial RANKL treatment or 3 days later and then replaced, with media changes every 2 days. Vehicle control samples were treated with 0.1% DMSO or ethanol.

### Detection of apoptosis

For survival analysis, preosteoclasts obtained by culturing mouse myeloid progenitors for 2 days in the presence of 50 ng/ml M-CSF and 50 ng/ml RANKL were suspended by 0.25% trypsin–EDTA (Sigma) and either analyzed immediately or cultured for an additional 12 or 18 hours in α-minimum essential medium in the absence of serum/cytokines. Cells were stained with phycoerythrin-conjugated annexin V and 7-aminoactinomycin D (7-AAD) (Apoptosis Detection Kit; BD PharMingen) according to the manufacturer's instructions and analyzed on a BD FACSCalibur flow cytometer.

For the TUNEL reaction, osteoclast cultures were stained with a Roche In Situ Cell Death Detection Kit, AP, according to the manufacturer's instructions. The number of TUNEL-positive cells was counted manually.

### Fluorescence microscopy

For F-actin staining, mouse myeloid progenitors were cultured for the indicated time periods in the presence of 50 ng/ml M-CSF and 50 ng/ml RANKL with or without the indicated inhibitors, fixed with 4% paraformaldehyde, permeabilized with 0.1% Triton X-100 (Sigma), and stained with 1:400 Alexa Fluor 488–conjugated phalloidin (Invitrogen) and 1:1,000 DAPI (Invitrogen). After several washes, fluorescence was observed using a Leica DMI6000B inverted microscope.

For live imaging of osteoclast development, myeloid progenitors obtained from Lifeact–EGFP–transgenic wild-type or PI3Kβ^−/−^ mice were cultured for the indicated time period in the presence of 50 ng/ml M-CSF and 50 ng/ml RANKL with or without the indicated inhibitors, and imaged using an Essen BioScience IncuCyte Zoom imaging system inside a tissue culture incubator or a Nikon BioStation IM-Q imaging system (Auro-Science Hungary). Videos were generated using IncuCyte Zoom Controller 2013A or Nikon BioStation IM software. For acidic vesicle staining, mouse myeloid progenitors cultured for 3 days in the presence of 50 ng/ml M-CSF and 50 ng/ml RANKL were incubated for 20 minutes with LysoTracker Red (Invitrogen) according to the manufacturer's instructions, then fixed, stained with DAPI, and imaged as described above.

### Analysis of gene expression

To test gene expression changes, mouse myeloid progenitors were cultured for 0–3 days in the presence of 50 ng/ml M-CSF with or without 50 ng/ml RANKL, followed by RNA extraction and reverse transcription as previously described (26,28). Quantitative reverse transcription–polymerase chain reaction was then performed using TaqMan assays for the mouse genes *Pik3ca* (Mm00435673_m1), *Pik3cb* (Mm00659576_m1), *Pik3cg* (Mm00445038_m1), and *Pik3cd* (Mm00435674_m1) and the osteoclast-specific genes *Acp5*, *Ctsk*, *Itgb3*, *Calcr*, *Nfatc1*, and *Tm7sf4* as previously described (26). Transcript levels relative to GAPDH were calculated using the comparative C_t_ method (26).

### Cathepsin K secretion

Mouse myeloid progenitors were cultured for 3 days in the presence of 50 ng/ml M-CSF and 50 ng/ml RANKL and then stimulated with 50 ng/ml phorbol myristate acetate (Sigma) for 10 minutes. The supernatants were collected, and the proteins were precipitated with acetone. Whole-cell lysates were obtained using Triton X-100–based lysis buffer (29). Samples were subjected to sodium dodecyl sulfate–polyacrylamide gel electrophoresis and immunoblotted with mouse monoclonal antibodies against cathepsin K (E-7; Santa Cruz Biotechnology) with secondary reagents from GE Healthcare.

### Micro–computed tomography (micro-CT) analysis

Trabecular bone structure and mineralization were tested by micro-CT analysis of the distal metaphysis of the femurs of age- and sex-matched wild-type and PI3Kβ^−/−^ mice essentially as described previously (26). Micro-CT sections were acquired using a SkyScan 1172 micro-CT apparatus with an isometric voxel size of 4.5 μm, followed by reconstitution of a horizontal section 250 sections proximal to the distal growth plate and reconstitution of a 3-dimensional axial cylinder 700 μm in diameter expanding from 150 to 450 sections proximal to the distal growth plate, as well as calculation of quantitative micro-CT parameters using SkyScan NRecon and CT-Analyser software (both from SkyScan) as described previously (26).

### Histomorphometric analysis

Histomorphometry studies were performed on the distal metaphysis of the femurs of age-matched wild-type and PI3Kβ^−/−^ male mice at ages 8–10 weeks. Bones were fixed, decalcified in 14% EDTA, embedded in paraffin, and sectioned and stained with TRAP, toluidine blue, and hematoxylin and eosin. Histomorphometric analysis was performed using a Zeiss Axioskop 2 microscope equipped with a video camera and an OsteoMeasure system (OsteoMetrics) according to international standards as described previously (30).

### Statistical analysis

All experiments were performed ≥3 times (or on ≥3 individual mice), with comparable results. Statistical analysis was performed using Student's unpaired 2-sample *t*-test.

## RESULTS

### PI3Kβ expression during osteoclast development

**Figure 1:**
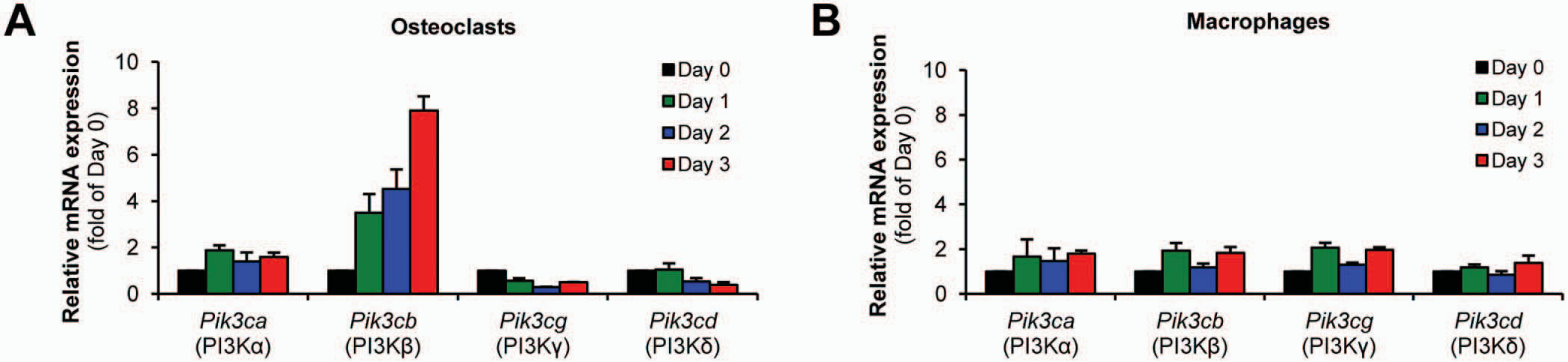
Expression of phosphoinositide 3-kinase β (PI3Kβ) during in vitro osteoclast development. Gene expression was analyzed in wild-type mouse myeloid precursors cultured for the indicated time periods in the presence of 50 ng/ml macrophage colony-stimulating factor with 50 ng/ml RANKL (osteoclasts) (A) or without RANKL (macrophages) (B). The expression of the *Pik3ca*, *Pik3cb*, *Pik3cg*, and *Pik3cd* (encoding the catalytic subunits of PI3Kα, PI3Kβ, PI3Kγ, and PI3Kδ, respectively) was determined by quantitative reverse transcription–polymerase chain reaction. Bars show the mean ± SEM from 3 independent experiments.

We first tested the expression of the various PI3K isoforms during in vitro differentiation of mouse progenitors in the presence of 50 ng/ml M-CSF with (osteoclasts) or without (macrophages) 50 ng/ml RANKL. As shown in Figure 1A, the expression of PI3Kβ but not of the other PI3K isoforms was dramatically up-regulated during osteoclast differentiation. In contrast, no substantial changes were seen in parallel macrophage cultures (Figure 1B). Therefore, consistent with a recent report (31), PI3Kβ was dramatically and specifically up-regulated during osteoclast development.

### Inhibition of PI3Kβ blocks osteoclast development and function in vitro

**Figure 2:**
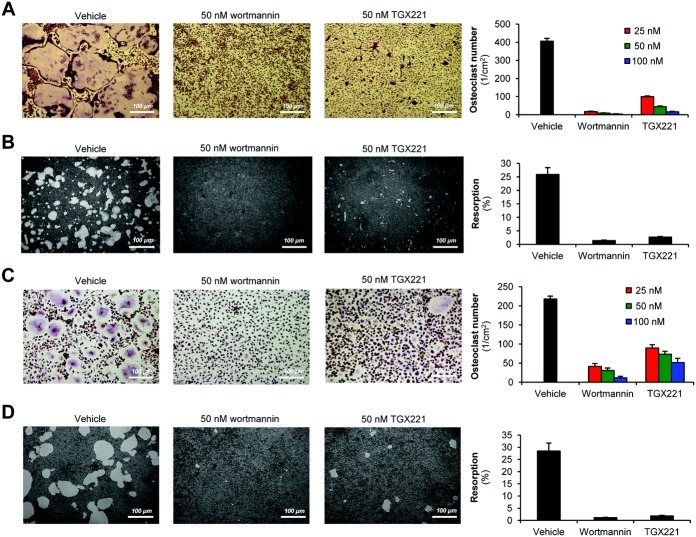
Pharmacologic inhibition of phosphoinositide 3-kinase β (PI3Kβ) blocks development and function of murine and human osteoclasts. Shown are representative images and quantification of tartrate-resistant acid phosphatase (TRAP)–stained cell cultures of (A and C), and in vitro resorption pit formation by (B and D) wild-type mouse bone marrow–derived osteoclasts (A and B) and human blood mononuclear cell–derived osteoclasts (C and D) cultured for 3 days (A), 11 days (B), or 14 days (C and D) in the presence of 50 ng/ml macrophage colony-stimulating factor, 50 ng/ml RANKL, and the indicated concentrations of PI3K inhibitors or 0.1% vehicle. Resorption pits appear as lighter areas. Osteoclasts are defined as TRAP-positive cells with ≥3 nuclei. In vitro resorption is defined as the percentage of resorbed area. Bars show the mean ± SEM from 3 independent experiments. Images are representative of 8–20 (A), 4–9 (B), or 3–4 (C and D) independent experiments.

To assess the functional role of PI3Kβ in osteoclasts, we next tested the effect of the PI3Kβ inhibitor TGX221 on in vitro–generated osteoclasts. As shown in Figure 2A, the nonselective PI3K inhibitor wortmannin completely blocked development of osteoclasts (large multinucleated TRAP-positive cells) generated by culturing mouse myeloid progenitors in the presence of 50 ng/ml M-CSF and 50 ng/ml RANKL. TGX221 also dose-dependently inhibited osteoclast development by ∼90% at a concentration of 50 n*M*, a dose thought to specifically inhibit PI3Kβ (8,32,33) (Figure 2) (further information is available at semmelweis.hu/elettan/en/?p=664).

We also tested the effect of TGX221 on the resorptive activity of osteoclasts. As shown in Figure 2B, 50 n*M* wortmannin abrogated the resorption of an artificial hydroxyapatite surface by murine osteoclast cultures generated using 50 ng/ml M-CSF and 50 ng/ml RANKL. TGX221 at 50 n*M* also nearly completely blocked the resorptive activity of osteoclasts under such conditions (Figure 2B).

We next tested the effect of TGX221 on human osteoclasts differentiated from PBMCs in the presence of 50 ng/ml human M-CSF and 50 ng/ml human RANKL. Both wortmannin and TGX221 strongly reduced the number of osteoclasts, with 50 n*M* TGX221 causing ∼70% inhibition (Figure 2C) (further information is available at semmelweis.hu/elettan/en/?p=664). In addition, 50 n*M* wortmannin or 50 n*M* TGX221 dramatically inhibited the in vitro resorptive capacity of human osteoclasts on an artificial hydroxyapatite layer (Figure 2D). Results similar to those described above were obtained when the concentration of M-CSF and RANKL was reduced to 20 ng/ml (further information is available at semmelweis.hu/elettan/en/?p=664). Taken together, these findings show that PI3Kβ likely plays an important role in the in vitro development and resorptive function of both human and mouse osteoclasts.

### In vivo bone homeostasis in PI3Kβ^−/−^ mice

**Figure 3:**
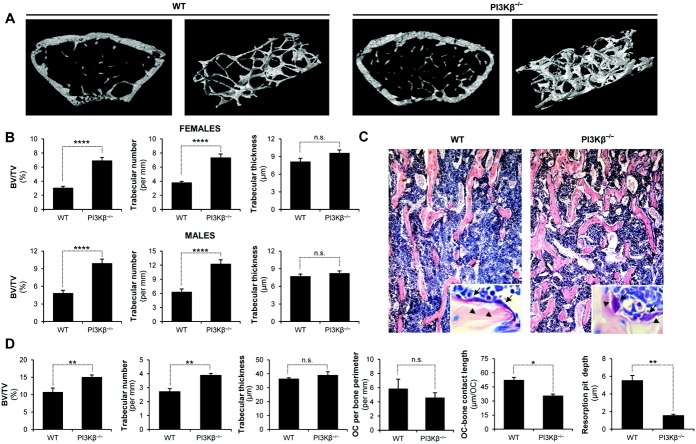
Phosphoinositide 3-kinase β (PI3Kβ) is required for in vivo bone homeostasis in mice. A, Representative single micro–computed tomography (micro-CT) cross-sections (left) and 3-dimensional reconstitution of an axial cylinder (right) of the trabecular area of the distal femoral metaphysis of 9-week-old wild-type (WT) and PI3Kβ^−/−^ female mice. B, Quantitative micro-CT analysis of the trabecular bone architecture of WT and PI3Kβ^−/−^ female and male mice ages 8–10 weeks. C, Representative images of histomorphometric analysis of the trabecular area of WT and PI3Kβ^−/−^ male mice at age 8 weeks. Original magnification × 10. Insets show enlarged view of tartrate-resistant acid phosphatase–stained sections with osteoclasts (arrows) and resorption pits (arrowheads). D, Histomorphometric analysis of the trabecular bone architecture and the number of osteoclasts (OC), the length of attachment of osteoclasts to the trabecular bone surface (OC bone contact length), and the depth of the resorption pits. Data were obtained from 8 (A and B) or 5 (C and D) mice per genotype. Bars show the mean ± SEM. * = *P* < 0.05; ** = *P* < 0.01; *** = *P* < 0.002; **** = *P* < 0.0004. BV/TV = bone volume/total volume; NS = not significant.

To test the role of PI3Kβ in osteoclast biology and bone homeostasis using a genetic approach, we turned to the analysis of PI3Kβ^−/−^ mice carrying a targeted deletion within the catalytic domain of PI3Kβ (22). We first analyzed trabecular bone structure using micro-CT analysis of the distal metaphysis of the femurs at ages 8–10 weeks. As shown in Figure 3A, significantly more trabeculae were seen in PI3Kβ^−/−^ mice, both in representative single micro-CT slices and in 3-dimensional reconstitution of an axial cylinder. Quantification of the entire trabecular area (Figure 3B) revealed significantly increased percent bone volume/total volume (BV/TV) in PI3Kβ^−/−^ mice in both females (*P* = 0.000029; n = 8) and males (*P* = 0.000084; n = 8), which was primarily due to increased trabecular number rather than increased thickness of the individual trabeculae. The increased BV:TV ratio was seen across all age groups tested (further information is available at semmelweis.hu/elettan/en/?p=664).

We also performed histologic and histomorphometric analysis of the trabecular bone of the distal femurs. Trabecular density was increased in PI3Kβ^−/−^ mice (Figure 3C); the quantitative BV:TV ratio (*P* = 0.0059; n = 5) and trabecular number, but not trabecular thickness, were also increased in PI3Kβ^−/−^ mice (Figure 3D).

We next analyzed osteoclasts visible in the TRAP-stained histologic sections. There was a moderate but not statistically significant (*P* = 0.44; n = 5) reduction in the average number of osteoclasts per bone perimeter in PI3Kβ^−/−^ mice (Figure 3D). Further analysis revealed that osteoclasts in PI3Kβ^−/−^ mouse sections were more rounded (Figure 3C, inset) with significantly shorter bone contact length (*P* = 0.030; n = 30 osteoclasts) (Figure 3D). In addition, the depth of resorption pits was dramatically reduced in PI3Kβ^−/−^ mouse sections (*P* = 0.0089; n = 30 osteoclasts) (Figures 3C [inset] and D). Taken together, these results indicate that PI3Kβ^−/−^ mice have increased trabecular bone volume, likely caused by moderately reduced numbers and abnormal morphology/function of osteoclasts.

### PI3Kβ deficiency impairs osteoclast development and function in vitro

**Figure 4:**
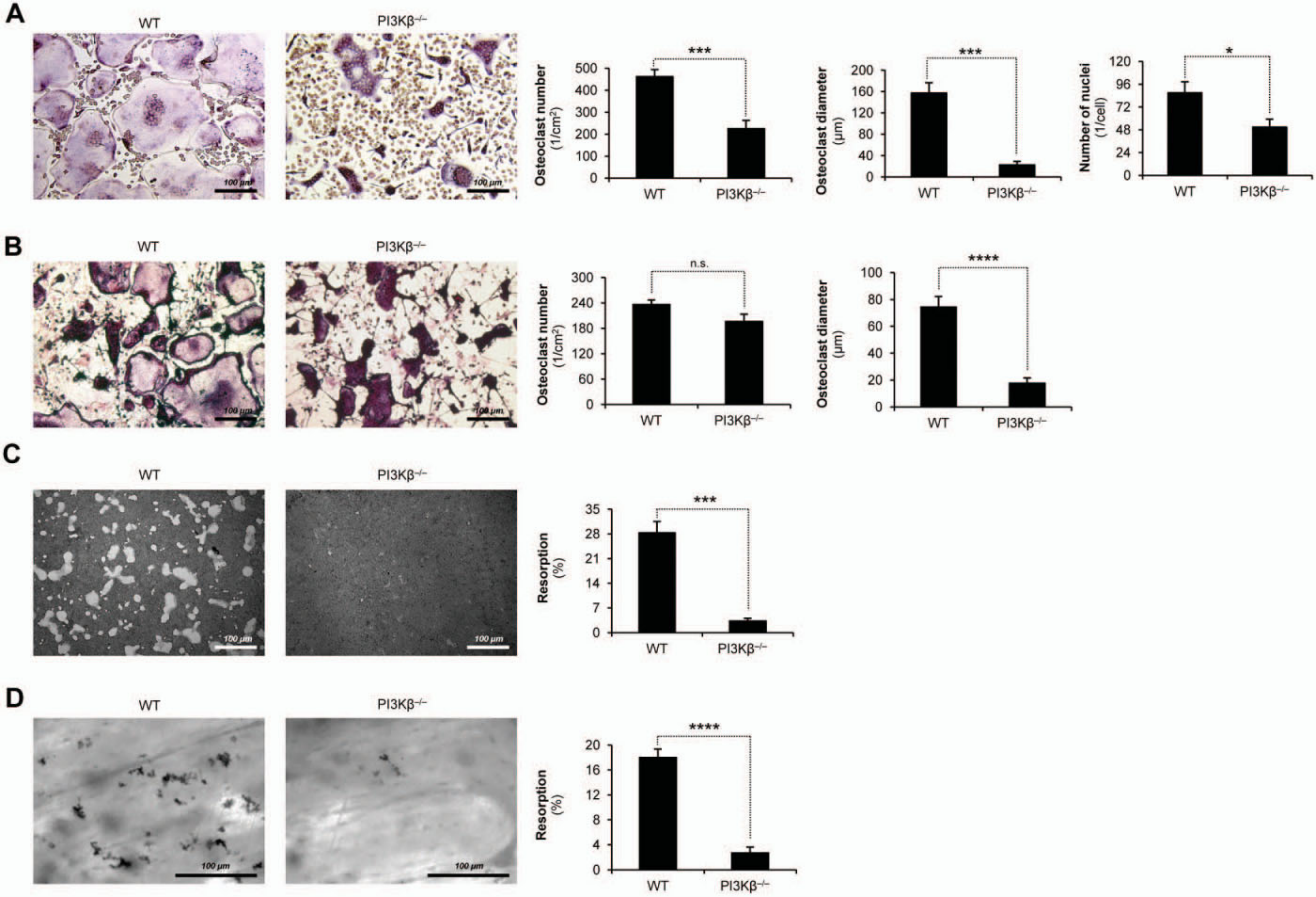
Genetic deficiency of PI3Kβ leads to defective osteoclast development and function. Shown are representative images and quantification of tartrate-resistant acid phosphatase–stained cell cultures of (A and B) and in vitro resorption pit formation on artificial hydroxyapatite (C) or on bovine bone slices (D) by, WT and PI3Kβ^−/−^ mouse bone marrow–derived osteoclasts cultured for 3 days (A), 10 days (B), or 11 days (C and D) in the presence of 50 ng/ml macrophage colony-stimulating factor and 50 ng/ml RANKL (A, C, and D) or with WT mouse calvarial osteoblasts (B). Bars show the mean ± SEM from 5–6 independent experiments. Images are representative of 8–10 (A), 3–4 (B), or 4–5 (C and D) independent experiments. * = *P* < 0.05; *** = *P* < 0.002; **** = *P* < 0.0004. See Figure 3 for definitions.

Next, we tested the effect of the PI3Kβ^−/−^ mutation on in vitro osteoclast development and function. As shown in Figure 4A, PI3Kβ deficiency led to reduced numbers of osteoclasts generated in the presence of 50 ng/ml M-CSF and 50 ng/ml RANKL (*P* = 0.0015; n = 5), and the average diameter of those cells was even more dramatically reduced (*P* = 0.00083; n = 5). Similar results were obtained when the concentration of M-CSF and RANKL was reduced to 20 ng/ml (further information is available at semmelweis.hu/elettan/en/?p=664). Fluorescence labeling of DNA (Figure 4A) also revealed significantly decreased numbers of nuclei per osteoclast (*P* = 0.018; n = 6).

We also tested osteoclast differentiation in osteoblast–osteoclast cocultures. We observed a moderate but not statistically significant (*P* = 0.12; n = 5) reduction of the number of osteoclasts differentiated from PI3Kβ^−/−^ mouse bone marrow cells. However, the diameter of osteoclasts was dramatically reduced in PI3Kβ^−/−^ mouse cell cultures (*P* = 0.00012; n = 5).

We next tested the resorptive activity of PI3Kβ^−/−^ mouse cell cultures. PI3Kβ deficiency strongly reduced the resorptive capacity of in vitro osteoclast cultures in the presence of 50 ng/ml M-CSF and 50 ng/ml RANKL, both on an artificial hydroxyapatite surface (*P* = 0.00042; n = 5) (Figure 4C) and on bovine bone slices (*P* = 6.2 × 10^−6^; n = 5) (Figure 4D). Results similar to those described above were obtained when the concentration of M-CSF and RANKL was reduced to 20 ng/ml (further information is available at semmelweis.hu/elettan/en/?p=664). Taken together, these findings demonstrate that genetic deficiency of PI3Kβ impairs in vitro development and resorptive function of osteoclasts.

### PI3Kβ is not required for osteoclast-specific gene expression

**Figure 5:**
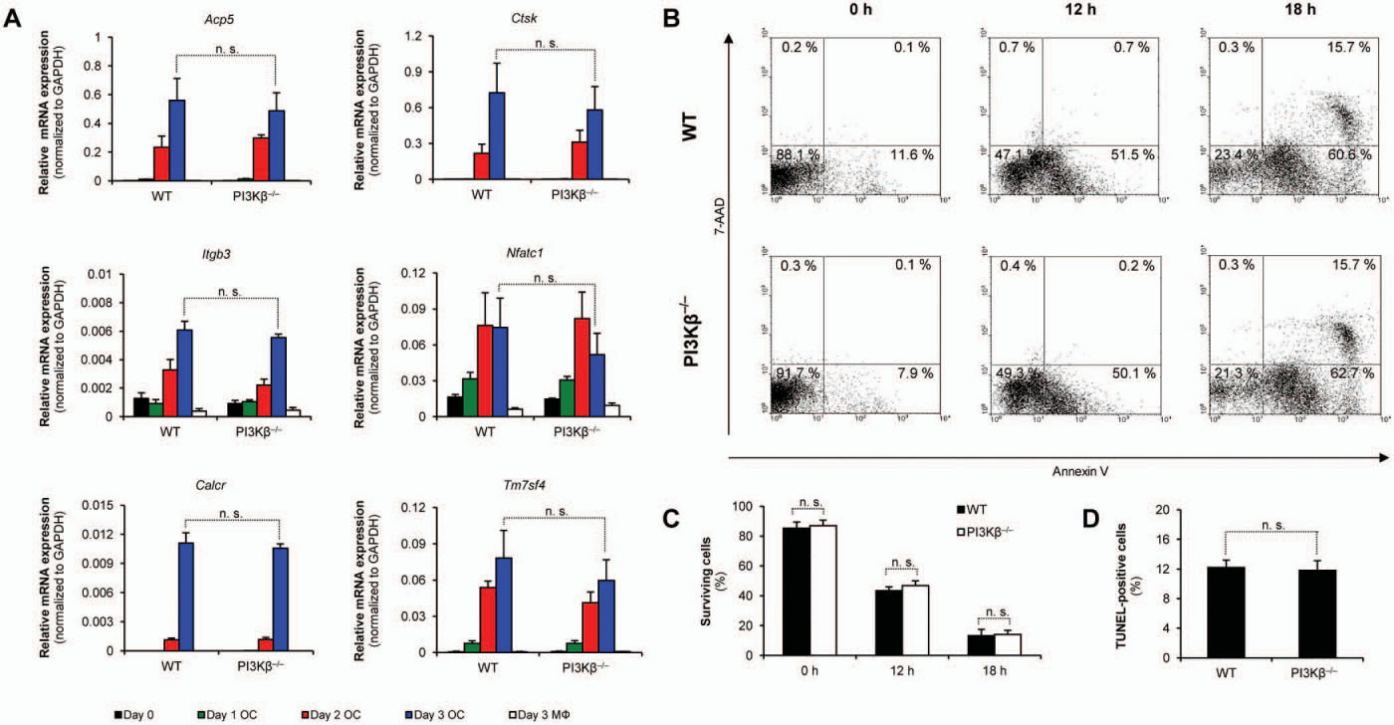
PI3Kβ is not required for up-regulation of osteoclast-specific gene expression and survival of osteoclasts. A, Gene expression in WT and PI3Kβ^−/−^ mouse bone marrow–derived cells cultured for the indicated time periods in the presence of 50 ng/ml macrophage colony-stimulating factor (M-CSF) with 50 ng/ml RANKL (osteoclasts; OC) or without RANKL (macrophages; MΦ). The expression of *Acp5*, *Ctsk*, *Itgb3*, *Nfatc1*, *Calcr*, and *Tm7sf4* (encoding tartrate-resistant acid phosphatase, cathepsin K, integrin β_3_ chain, NF-ATc1, calcitonin receptor, and dendritic cell–specific transmembrane protein, respectively) was determined by quantitative reverse transcription–polymerase chain reaction. B and C, Representative flow cytometric profiles (B) and quantification (C) of the binding of phycoerythrin (PE)–conjugated annexin V (apoptosis marker) and 7-aminoactinomycin D (7-AAD) (necrosis marker) to WT and PI3Kβ^−/−^ mouse preosteoclasts (generated by culturing myeloid precursors in the presence of 50 ng/ml M-CSF and 50 ng/ml RANKL for 2 days) before (0 hours) or after serum and cytokine starvation for 12 or 18 hours. Surviving cells are defined as negative for both PE-conjugated annexin V and 7-AAD staining. D, Analysis of the number of TUNEL-positive cells in WT and PI3Kβ^−/−^ mouse bone marrow–derived osteoclast cultures. Data are from 3 (A) or 5-6 (B–D) independent experiments. Bars show the mean ± SEM. See Figure 3 for other definitions.

To better understand the role of PI3Kβ in osteoclasts, we next tested the expression of osteoclast-specific genes during differentiation of murine myeloid progenitors in the presence of 50 ng/ml M-CSF and 50 ng/ml RANKL (osteoclasts) or M-CSF alone (macrophages). As shown in Figure 5A, the expression of *Acp5* (encoding TRAP), *Ctsk* (encoding cathepsin K), *Itgb3* (encoding integrin β_3_ chain), *Nfatc1* (encoding NF-ATc1), *Calcr* (encoding calcitonin receptor), and *Tm7sf4* (encoding dendritic cell–specific transmembrane protein) was strongly increased during osteoclast differentiation but not during macrophage differentiation. PI3Kβ deficiency did not affect the expression of any of those genes (Figure 5A), indicating that PI3Kβ is not required for osteoclast-specific gene expression.

### Normal survival and apoptosis of PI3Kβ^−/−^ mouse osteoclast-lineage cells

To test the role of PI3Kβ deficiency in survival and apoptosis of osteoclast-lineage cells, preosteoclasts were generated by culturing myeloid progenitors in 50 ng/ml M-CSF and 50 ng/ml RANKL for 2 days, followed by withdrawal of serum and cytokines for 12 or 18 hours. As shown in Figures 5B and C, ∼90% of the preosteoclasts before serum/cytokine withdrawal (0-hour samples in Figure 5B) were negative for the apoptosis marker annexin V and the necrosis marker 7-AAD, whereas serum/cytokine withdrawal triggered apoptosis and then necrosis of the cells. No difference in any of those processes was observed between cultures of cells from wild-type and PI3Kβ^−/−^ mice (Figures 5B and C).

We also tested apoptosis in regular osteoclast cultures by in situ TUNEL staining. As shown in Figure 5D, ∼12% of cells were TUNEL positive in cultures of osteoclasts from both wild-type and PI3Kβ^−/−^ mice. Taken together, the observations suggest that PI3Kβ deficiency does not affect survival, apoptosis, or necrosis of osteoclast-lineage cells.

### PI3Kβ is required for actin ring formation

**Figure 6:**
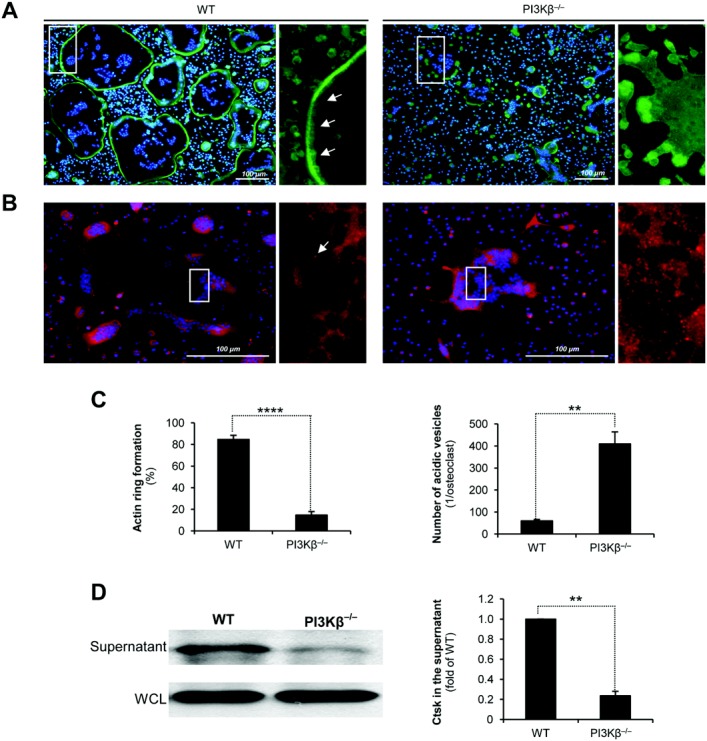
PI3Kβ is required for actin ring formation and cathepsin K secretion by murine osteoclasts. A and C (left), Representative fluorescence images (A) and quantification (C [left]) of WT and PI3Kβ^−/−^ mouse myeloid precursors cultured in the presence of 50 ng/ml macrophage colony-stimulating factor and 50 ng/ml RANKL for 3 days and then stained with Alexa Fluor 488–conjugated phalloidin and DAPI. Arrows in A show the actin ring. Values in C (left) are the percentage of osteoclasts with closed actin rings at the cell periphery. B and C (right), Representative fluorescence images (B) and quantification (C [right]) of WT and PI3Kβ^−/−^ mouse myeloid precursors cultured under conditions similar to those described in A and then stained with LysoTracker Red and DAPI. An acidic vesicle is marked with an arrow in B. Values in C (right) are the number of acidic vesicles. Boxed areas at left in A and B are shown at higher magnification at right. D, Immunoblot (left) and densitometric quantification (right) of cathepsin K (Ctsk) in the supernatant or whole-cell lysates (WCL) of WT and PI3Kβ^−/−^ mouse bone marrow cells cultured as described in A. Fluorescence images are representative of 4–6 independent experiments; immunoblot is representative of 4 independent experiments. Bars show the mean ± SEM from 3–6 independent experiments. ** = *P* < 0.01; **** = *P* < 0.0004. See Figure 3 for other definitions.

We next tested whether PI3Kβ is required for the formation of the osteoclast actin ring, which is likely involved in sealing the resorption pit. A continuous F-actin ring was observed at the periphery of most wild-type mouse osteoclasts in the presence of 50 ng/ml M-CSF and 50 ng/ml RANKL (Figures 6A and C). In contrast, even multinucleated cells in PI3Kβ^−/−^ mouse osteoclast cultures rarely showed a clear F-actin ring, and F-actin was instead accumulated in the cytoplasm and distinct patches at the cell periphery (*P* = 7.8 × 10^−8^; n = 6).

We also assessed temporal changes of actin polymerization using transgenic mice expressing EGFP-tagged Lifeact, a short peptide specifically binding to fibrillar actin. As shown in Supplementary Videos 1 and 2 (available on the *Arthritis & Rheumatology* web site at http://onlinelibrary.wiley.com/doi/10.1002/art.38660/abstract) (further information is available at semmelweis.hu/elettan/en/?p=664), osteoclasts derived from Lifeact–EGFP–expressing wild-type mouse progenitor cells began to form continuous F-actin rings ∼2 days after addition of RANKL, and these remained visible until the osteoclasts succumbed to apoptosis. In contrast, osteoclast-like cells from Lifeact–EGFP–transgenic PI3Kβ^−/−^ mice failed to form actin rings and instead showed patchy/dispersed Lifeact distribution. Taken together, these findings indicate that PI3Kβ plays an important role in the formation of the osteoclast actin ring.

### PI3Kβ^−/−^ mouse osteoclasts retain acidic vesicles and fail to release cathepsin K

During bone resorption, osteoclasts deliver and exocytose lysosome-related, cathepsin K–containing acidic vesicles to the ruffled border (34). Since prior studies proposed a role for PI3K activity in this process (31,35), we tested the distribution of acidic vesicles in PI3Kβ^−/−^ mouse cell cultures in the presence of 50 ng/ml M-CSF and 50 ng/ml RANKL. While wild-type mouse osteoclasts contained very few small acidic vesicles, PI3Kβ^−/−^ mouse cells were fully packed with such vesicles, suggesting that they cannot be discharged and therefore are retained in PI3Kβ^−/−^ mouse cells (*P* = 0.0021; n = 5) (Figures 6B and C).

Exocytosis of the matrix-degrading enzyme cathepsin K plays an important role in osteoclast-mediated bone resorption. As shown in Figure 6D, the PI3Kβ^−/−^ mutation substantially reduced the amount of cathepsin K in the supernatant of osteoclast cultures (*P* = 0.0064; n = 3). Taken together, these results show that the absence of PI3Kβ leads to defective actin ring formation, accumulation of acidic vesicles, and impaired release of cathepsin K into the extracellular space.

### Inhibition of PI3Kβ after osteoclast formation blocks resorption and actin ring maintenance

The above results suggested that PI3Kβ not only is involved in osteoclast development but also plays important functional roles in mature osteoclasts. To test that possibility, we investigated the effect of PI3Kβ inhibition after the formation of mature osteoclasts. As shown at semmelweis.hu/elettan/en/?p=664, adding 50 n*M* TGX221 to osteoclast cultures starting from 3 days after the initial RANKL treatment strongly inhibited resorption of an artificial hydroxyapatite layer (*P* = 0.0093; n = 3). As shown at semmelweis.hu/elettan/en/?p=664, treatment of osteoclasts with 50 n*M* TGX221 for 6 hours starting 3 days after the initial RANKL treatment (when mature osteoclasts with complete actin rings have already been formed) led to the disappearance of actin rings (*P* = 2.4 × 10^−4^; n = 3). Kinetic analysis of the effect of TGX221 using Lifeact–EGFP–expressing cells revealed that the actin rings began to resolve immediately after TGX221 treatment and became highly fragmented within a few hours (further information is available at semmelweis.hu/elettan/en/?p=664) (see Supplementary Videos 3 and 4, available on the *Arthritis & Rheumatology* web site at http://onlinelibrary.wiley.com/doi/10.1002/art.38660/abstract). Taken together, these observations suggest that inhibition of PI3Kβ blocks the resorptive function and actin ring maintenance of in vitro osteoclast cultures even if performed several days after initial RANKL treatment.

## DISCUSSION

Recent studies revealed highly specific functions of the various PI3K isoforms, triggering development of isoform-specific PI3K inhibitors for therapeutic purposes (2,36). Although prior studies suggested a role for PI3K activity in osteoclast development and bone resorption (18–21), little is known about the specific PI3K isoform(s) participating in those responses. Our initial gene expression studies (Figure 1) suggested a functional role for PI3Kβ in osteoclasts, which was tested using a detailed genetic and pharmacologic approach. Those studies revealed a critical role for PI3Kβ in in vitro development and resorbing function of primary human and mouse osteoclasts (Figures 2 and 4) and in in vivo bone homeostasis in experimental mice (Figure 3).

Reduced osteoclast-mediated bone resorption may be due to failure of osteoclast development or defective resorptive function of mature osteoclasts. While PI3Kβ appears to play a role in osteoclast development under certain conditions (Figures 2A and C, Figures 4A and B), the most dramatic in vitro effects of PI3Kβ inactivation were observed when testing resorptive function or cellular parameters related to bone resorption (Figures 2, 4, and 6). Osteoclasts from PI3Kβ^−/−^ mice also showed reduced bone contact length and resorption pit depth in vivo (Figure 3D). Furthermore, addition of TGX221 to cultures with mature osteoclasts inhibited bone resorption and led to rapid disassembly of preexisting actin rings (further information is available at semmelweis.hu/elettan/en/?p=664). Those results suggest that the most critical role for PI3Kβ is to mediate the resorptive function of mature osteoclasts.

We recently reported the analysis of phospholipase Cγ2–deficient (PLCγ2^−/−^) mouse osteoclast cultures and bone morphology (26). It is interesting to note that while PI3Kβ^−/−^ and PLCγ2^−/−^ mice had similar increases in trabecular bone mass, PLCγ2^−/−^ mouse cells showed a much more robust in vitro osteoclast developmental defect than PI3Kβ^−/−^ mouse cells. Those results again suggest that the primary defect in PI3Kβ^−/−^ mice lies in the bone-resorbing function of osteoclasts.

The exact position of PI3Kβ in osteoclast signal transduction pathways is at present poorly understood. Osteoclast development and function are triggered through a large number of extracellular signals (9–11,16,17). Our initial experiments suggested that PI3Kβ is required for phosphorylation of Akt in response to M-CSF treatment (data not shown). However, a more detailed analysis of signaling by the various cell surface receptors and downstream signaling intermediates, as well as high-resolution imaging of phosphatidylinositol 3,4,5-trisphosphate generation in osteoclast cultures, would be needed to define the exact position of PI3Kβ in intracellular signaling of osteoclasts. Nevertheless, our findings indicate that PI3Kβ activation participates in reorganization of the actin cytoskeleton and release of cathepsin K–containing acidic vesicles (Figure 6) but not in gene expression changes or survival/apoptosis of the cells (Figure 5).

The results of prior studies using nonspecific PI3K inhibitors suggested a role for PI3Ks in osteoclast development and function (18–21). A few reported attempts to narrow the list of PI3K isoform(s) involved failed to reveal a major role for any of the specific PI3K isoforms, showed contradictory results, and were even inconsistent with a role for PI3Kβ (31,37,38). Our substantially more detailed experiments firmly identify PI3Kβ as a critical and likely predominant PI3K isoform involved in osteoclast development and function.

There is substantial interest in the pharmaceutical industry in the development of isoform-specific PI3K inhibitors for the treatment of various human diseases (1,3–5). Based on its role in platelet activation (32,39–43), PI3Kβ has been proposed to be a suitable target for the treatment of thrombotic diseases (44). Based on the results presented here, PI3Kβ may also be a suitable therapeutic target in diseases characterized by excessive osteoclast-mediated bone resorption such as rheumatoid arthritis, osteoporosis, or cancer-induced focal bone loss.
